# Genomic characterization of co-existing neoplasia and carcinoma lesions reveals distinct evolutionary paths of gallbladder cancer

**DOI:** 10.1038/s41467-021-25012-9

**Published:** 2021-08-06

**Authors:** Jianzhen Lin, Xinxin Peng, Kun Dong, Junyu Long, Xuejiao Guo, Hongyue Li, Yi Bai, Xu Yang, Dongxu Wang, Xin Lu, Yilei Mao, Xinting Sang, Xuwo Ji, Haitao Zhao, Han Liang

**Affiliations:** 1 Department of Liver Surgery, State Key Laboratory of Complex Severe and Rare Diseases, Peking Union Medical College Hospital, Chinese Academy of Medical Sciences & Peking Union Medical College (CAMS & PUMC), Beijing, China; 2 Pancreas Center, The First Affiliated Hospital of Nanjing Medical University, Pancreas Institute, Nanjing Medical University, Nanjing, China; 3 Precision Scientific (Beijing) Co. Ltd., Beijing, China; 4 Key Laboratory of Carcinogenesis and Translational Research (Ministry of Education), Department of Pathology, Peking University Cancer Hospital & Institute, Beijing, China; 5 Department of Bioinformatics and Computational Biology, The University of Texas MD Anderson Cancer Center, Houston, TX USA; 6 Department of Systems Biology, The University of Texas MD Anderson Cancer Center, Houston, TX USA

**Keywords:** Cancer genomics, Gall bladder cancer, Computational biology and bioinformatics

## Abstract

Gallbladder carcinoma is the most common cancer of the biliary tract with dismal survival largely due to delayed diagnosis. Biliary tract intraepithelial neoplasia (BilIN) is the common benign tumor that is suspected to be precancerous lesions. However, the genetic and evolutionary relationships between BilIN and carcinoma remain unclear. Here we perform whole-exome sequencing of coexisting low-grade BilIN (adenoma), high-grade BilIN, and carcinoma lesions, and normal tissues from the same patients. We identify aging as a major factor contributing to accumulated mutations and a critical role of *CTNNB1* mutations in these tumors. We reveal two distinct carcinoma evolutionary paths: carcinoma can either diverge earlier and evolve more independently or form through the classic adenoma/dysplasia-carcinoma sequence model. Our analysis suggests that extensive loss-of-heterozygosity and mutation events in the initial stage tend to result in a cancerous niche, leading to the subsequent BilIN-independent path. These results reframes our understanding of tumor transformation and the evolutionary trajectory of carcinogenesis in the gallbladder, laying a foundation for the early diagnosis and effective treatment of gallbladder cancer.

## Abstract (web summary)

The progression from biliary tract intraepithelial neoplasia (BilIN) to gallbladder carcinoma (GBC) remains unclear. Here the authors use genomics to analyze coexisting GBC lesions, low-grade and high-grade BilINs, revealing two distinct evolutionary paths for GBC development.

## Introduction

Gallbladder carcinoma (GBC) is the most common cancer of the biliary tract^1,2^. Its incidence rate shows a wide geographic variance, and in some countries such as Chile and India, the incidence rate is nearly 30/100000 in women^3^. Unlike other cancer types, only 30% of GBC patients are diagnosed or suspected preoperatively, whereas other cases are diagnosed based on postoperative incidental findings^1^. In most patients, the tumor is recognized either at the time of surgery or by the pathologist examining the surgical specimen. Most symptomatic GBC patients have an incurable tumor, and the risk of GBC recurrence is 64% at 5 years, even after R0 resection. The clinical outcome of GBC is very poor: although a 5-year survival rate of 75% can be achieved in early-stage (T1) disease, the overall 5-year survival rate is <5%^4^. Therefore, there is an urgent clinical need to identify high-risk patients and have their gallbladders removed before the development of GBC.

When considering the potential risk for gallbladder cancer, it is important to understand the relationship between gallbladder polypoid lesions and GBC, thereby managing them wisely and timely. The population prevalence of gallbladder polyps is ~5%, accounting for 2–12% of the cholecystectomy specimens^5,6^. Low-grade biliary tract intraepithelial neoplasia (LG-BilIN) and high-grade biliary tract intraepithelial neoplasia (HG-BilIN), two common benign neoplastic polyps, have attracted much attention due to their proposed premalignant behaviors. Specifically, morphological studies suggest the histologic transition of LG-BilIN (adenoma) into GBC, with or without neighboring HG-BilIN^7^. Molecular analyses show that p53 and *KRAS* are commonly mutated in LG-BilIN and GBC^8^, and the dysregulation of p16/cyclin-D1/CDK4 cell cycle pathway is associated with both gallbladder dysplasia and cancer cells^9^. Furthermore, there is a progressive increase in the average age of patients with LG-BilIN, LG-BilIN with malignant changes, and invasive GBC^10,11^. These observations suggest that LG-BilIN is a precancerous lesion that gradually progresses to GBC through a step-wise evolution, which is roughly analogous to colorectal carcinogenesis from adenoma^12^. However, some histological and genomic observations challenge the LG-BilIN-GBC sequential model. The most direct evidence is the relative rarity of LG-BilIN as compared to the frequency of GBC in patients undergoing cholecystectomy^13^. Also, LG-BilIN generally accompanies HG-BilIN rather than GBC^14^. Morphologically, it is uncommon for advanced GBC foci to co-exist in the vicinity of LG-BilIN, even for early or well-differentiated cancer cells^10^. HG-BilIN, rather than LG-BilIN, exhibits morphological features that are thought to facilitate progression to an infiltrating GBC^15^. The altered signaling pathways in LG-BilIN are distinct from those altered in GBC^16^. Additionally, the *KRAS* codon-12 mutation is detected more frequently in the de novo gallbladder carcinomas compared to carcinoma-in-pyloric-gland-type LG-BilIN^17^. Thus, the genetic and evolutionary relationships between LG-BilIN, HG-BilIN, and GBC, and the molecular events driving gallbladder carcinogenesis, remain unclear^18^. A comprehensive characterization of neighboring LG-BilIN, HG-BilIN, and GBC coexisting in the same patients will provide a unique opportunity to elucidate their evolutionary relationships by removing inter-tumor heterogeneity, a major confounding factor in previous studies^7,19,20^.

In this work, we perform laser microdissections on tissue sections to isolate tissues of the normal gallbladder, LG-BilIN, HG-BilIN, and GBC from the same individuals. Using whole-exome sequencing, we systematically investigated the GBC genomic landscape and the evolution of the carcinoma from a precancerous stage to GBC. Our study provides insights into the biology of gallbladder tumors and their evolutionary trajectories.

## Results

### Genomic characteristics of BilIN-related gallbladder carcinoma

**Fig. 1:**
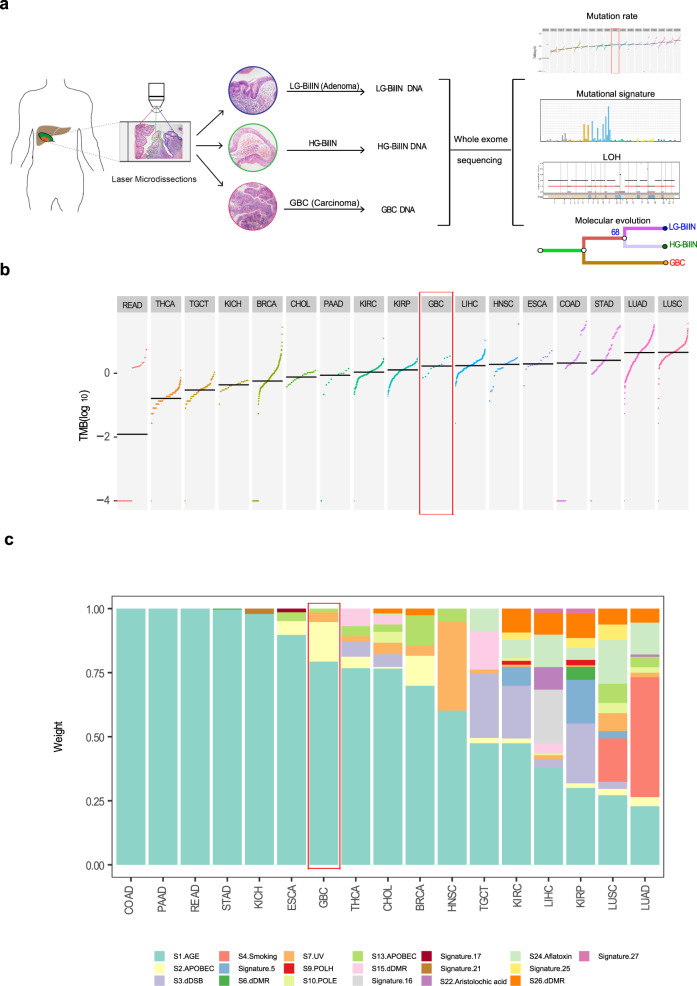
Study overview and mutation characteristics of BilIN-related GBC. **a** Overall study design. **b** The TMB distribution of BilIN-related gallbladder carcinoma (GBC) in the context of stage-I samples from 16 TCGA cancer types. **c** Relative contributions of different mutational signatures (annotated by etiology factors) in GBC and 16 TCGA cancer types. **b**, **c** GBC is highlighted in the red box. READ: rectum adenocarcinoma; THCA: thyroid carcinoma; TGCT: testicular germ cell tumors; KICH: kidney chromophobe; BRCA: breast invasive carcinoma; CHOL: cholangiocarcinoma; PAAD: pancreatic adenocarcinoma; KIRC: kidney renal cell carcinoma; KIRP: kidney renal papillary cell carcinoma; LIHC: liver hepatocellular carcinoma; HNSC: head and neck squamous cell carcinoma; ESCA: esophageal carcinoma; COAD: colon adenocarcinoma; STAD: stomach adenocarcinoma; LUAD: lung adenocarcinoma; LUSC: lung squamous cell carcinoma.

After a rigorous pathological review of a large number of gallbladder tumor samples, we collected tissue samples, including normal ones, from 11 patients diagnosed with T1 tumors, in whom GBC, LG-BilIN (gallbladder adenoma with low-grade dysplasia), and HG-BilIN (high-grade dysplasia or carcinoma in situ) lesions geographically coexisted (average age, 62). Despite the limited sample size, we noted more female patients in our cohort (female: 9; male: 2), which echoes the gender bias uncovered in epidemiological studies^21^. Age at diagnosis for nine of the 11 enrolled patients was above 60 years, except for patients P03 and P11, both diagnosed at 38. Through laser microdissection, we separated GBC, LG-BilIN, HG-BilIN, and normal gallbladder tissues and performed whole-exome sequencing on 44 samples in total (mean depth: normal 107×; GBC 171×; LG-BilIN 188×; HG-BilIN 206×) (Fig. 1a and Supplementary Data 1). We employed a well-established, multi-caller-based MC3 approach to call single nucleotide variant (SNV) mutations and small indels, and this approach has been used to generate the high-quality mutation data of The Cancer Genome Atlas (TCGA)^22^. Importantly, we performed a rescue step to call mutations across different samples from the same patient to reduce false negatives. Across the 33 tumor samples from the 11 patients, we identified an average of 214 SNVs (range: 69-725) and 9 indels (range: 3-48) (Supplementary Fig. 1a and Supplementary Data 2). To assess the accuracy of our mutation calls, we performed independent Sanger sequencing and reached a validation rate of 95% (42 out of 44 mutations) (Supplementary Fig. 2 and Supplementary Data 3). To assess the relative mutation burden of GBC, we compared our GBC samples with stage-I samples of 16 other cancer types from TCGA and found that GBC exhibited a moderate level of tumor mutation burden (TMB), similar to that of liver hepatocellular carcinoma (LIHC) (*t*-test, *p* = 0.79) but significantly higher than that of cholangiocarcinoma (CHOL) (*t*-test, *p* = 7.3 × 10^−4^, Fig. 1b).

In order to identify the mutagenesis mechanisms underlying the mutations and evolution of these GBC samples and elucidate the common/specific contributing factors, we decomposed the mutational spectrum for the 16 TCGA cancer types (stage-I only) as well as our GBC samples, based on 30 established mutational signatures (COSMIC mutational signature v2)^23^. This analysis confirmed some known signature patterns such as smoking (Signature 4) for lung cancers (Fig. 1c). We found that in most cancer types, the mutations in stage-I tumors were mainly caused by age, especially for colorectal cancer (COAD/READ), pancreatic adenocarcinoma (PAAD), and stomach cancer (STAD). In the GBC samples, age-related Signature 1 contributed to >75% of the mutations and APOBEC-related Signature 2 contributed ~10% (Fig. 1c). These results indicate the importance of mutations accumulated by the aging process for GBC development and provide the first view of mutational characteristics of GBC coexisting with LG/HG-BilIN.

### Somatic alteration feature comparison of gallbladder tumors

**Fig. 2:**
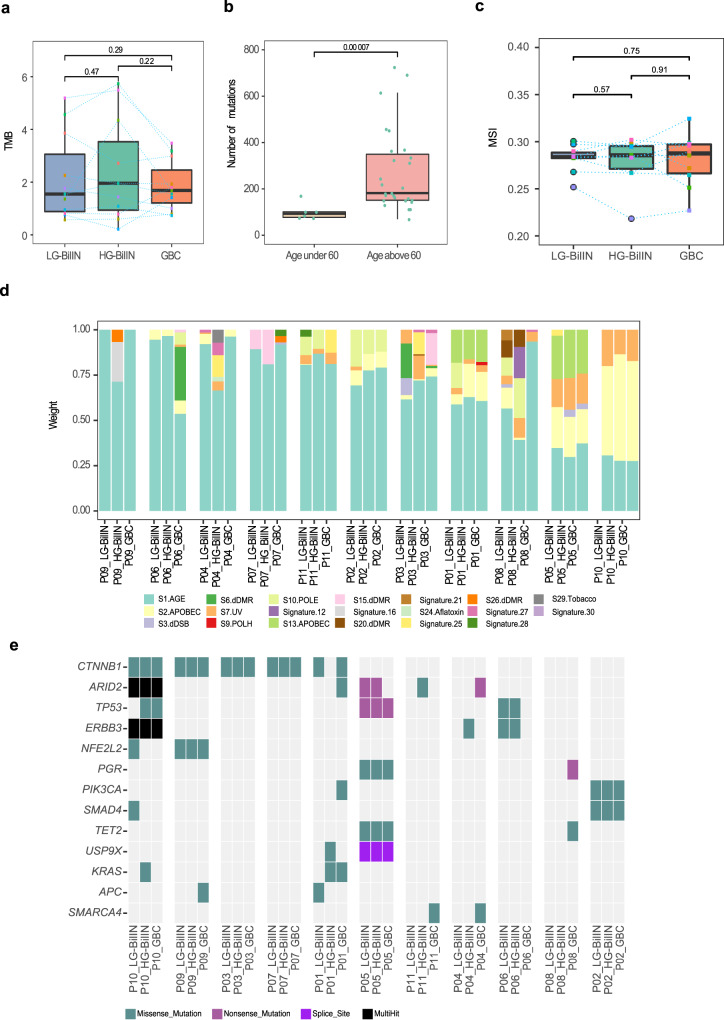
Mutational feature comparison among three types of gallbladder tumors. **a** TMB distribution per tumor type (*n* = 11 samples). **b** The number of mutations in tumor samples from young (*n* = 6 samples) and old patients (*n* = 27 samples). **c** MSI score distribution per tumor type (*n* = 10 samples). **d** Relative contributions of different mutational signatures (annotated by etiology factors) in tumor samples based on COSMIC mutational signature v2. **e** An oncoplot showing potential driver mutations detected in at least two patients. Mutations are colored according to different substitution types. **a**–**c** The middle line in the box is the median, the bottom, and top of the box are the first and third quartiles, and the whiskers extend to 1.5× interquartile range of the lower and the upper quartiles, respectively. Raw *p*-values based on two-tailed Student’s *t*-test are shown.

In addition to the above-mentioned comparison among malignant tumor types, we compared the mutational features among the three gallbladder tumor types. We found that GBC, LG-BilIN, and HG-BilIN showed similar mutation rates (paired *t*-test, *p* > 0.05, Fig. 2a), but the tumor samples from the two young patients (P03 and P11) showed much fewer mutations than those from older patients, regardless of tumor types (mean number, 101 vs. 273, *t*-test, *p* < 7 × 10^−5^, Fig. 2b). We further quantified the microsatellite instability (MSI) status of these tumor samples based on a large set of selective microsatellite loci^24^ but did not find a significant difference among tumor types (paired *t*-test, *p* > 0.05, Fig. 2c). The three tumor types also showed similar compositions in terms of six possible substitution classes (Supplementary Fig. 1b). Based on COSMIC mutational signature v2, different tumors from the same patient generally showed similar mutation signature patterns, but the signature composition varied considerably from patient to patient (Fig. 2d), suggesting that the underlying mutagenesis mechanisms are more patient-specific than tumor type-specific. Across the 33 tumor samples, there was a consistent component of age-related Signature 1. In addition, tumor samples from patient P10 showed APOBEC-related Signature 2 and ultraviolet exposure related Signature 7; tumors from patient P05 exhibited APOBEC-related signatures (Signature 2 and Signature 13) and Signature 7 (although Signature 7 is found predominantly in skin, head and neck or oral squamous cancers, it has been identified in other cancer types^25^). Tumors from P02 showed a sizable contribution from POLE-related Signature 10, which was further confirmed by the mutational analysis based on the COMSIC mutation signature v3 (Supplementary Fig. 3). The GBC samples from P03, P06, and P08 showed some signature divergences from their corresponding benign tumors, LG-BilIN, and HG-BilIN.

To understand key somatic mutations during tumor development, we examined the potential somatic mutation drivers. To overcome the relatively small sample size of our cohort, we focused on known drivers identified in a recent TCGA pan-cancer analysis^26^. In total, there were 16 potential mutation drivers observed in at least two patients. Among them, mutations in *CTNNB1* and *ARID2* were detected in five out of the 11 patients, and *TP53* and *ERBB3* mutations were identified in three patients (Fig. 2e). GBC samples did not contain more driver mutations than LG- or HG-BilIN. As for specific mutations, five patients harbored four different *CTNNB1* mutations, including p.T41I shared by all three tumor samples from P03, p.S45F shared in P07 and P10, p.S33C in P09, and p.S45F and p.K335T in LG-BilIN and GBC from P01, respectively. All these *CTNNB1* mutations were probably damaging, as suggested by a PolyPhen^27^ score of >0.9. *CTNNB1* mutations have been reported to cause modified β-catenin activity associated with liver tumor progression^28^. This observation highlights the key role of β-catenin activity driven by *CTNNB1* mutations for cellular transformation in GBC tumorigenesis. For mutations in *ARID2*, a subunit of the PBAF chromatin-remodeling complex, five patients harbored six mutations: only p.Q916* was shared by the three tumor samples from P10, suggesting loss-of-function *ARID2* as a driver in this patient; in the other four patients, *ARID2* mutations were only detected in GBC or BilIN samples. Among the three patients harboring *ERBB3* mutations, one mutation was shared in all three samples of P10, and the other two patients (P04 and P06) only contained a mutation in BilIN samples. We observed *SMARCA4* mutations in the GBC samples but not in BilIN samples from P04 and P11, suggesting that those mutations were acquired in a late stage in GBC development. We also investigated the somatic copy-number alterations (SCNAs) inferred from WES data across the 33 tumor samples. Among the 11 GBC samples, we noted significant copy-number gain of *ERBB2* (four samples) and significant copy-number loss of *CDNK2A* (four samples). These SCNAs may have played a role in the GBC tumorigenesis (Supplementary Fig. 4). Altogether, these results provide a shortlist of driver somatic alteration candidates in the development of adenoma/dysplasia-related GBC.

### Two GBC evolutionary paths driven by early LOH and mutation loads

**Fig. 3:**
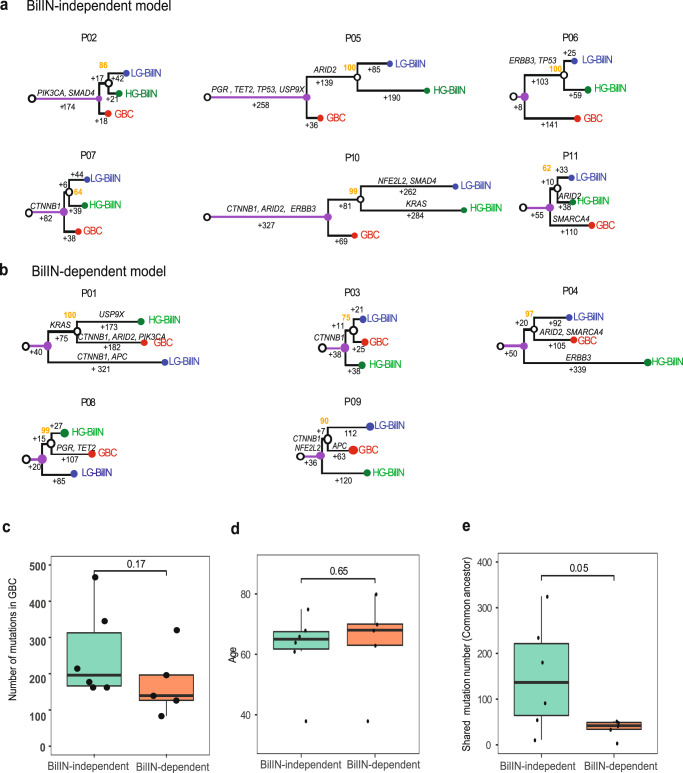
Phylogenetic trees of gallbladder tumors. **a** BilIN-independent group (*n* = 6), and **b** BilIN-dependent group (*n* = 5). The branch lengths are proportional to the number of supporting mutations, which were labeled. Potential driver mutations are annotated along the branches. Bootstrap values are shown in orange. **c** The number of mutations in GBC samples (BilIN-independent: *n* = 6; BilIN-dependent: *n* = 5), **d** age at diagnosis (BilIN-independent: *n* = 6; BilIN-dependent: *n* = 5), and **e** the mutations accumulated in the common ancestor state of LG-BilIN/HG-BilIN/GBC, in the BilIN-independent and BilIN-dependent groups (BilIN-independent: *n* = 6; BilIN-dependent: *n* = 5). The middle line in the box is the median, the bottom, and top of the box are the first and third quartiles, and the whiskers extend to 1.5× interquartile range of the lower and the upper quartiles, respectively. Raw *p*-values based on the two-tailed Wilcoxon rank-sum test are shown.

Somatic mutations provide a molecular footprint for cancer evolution. Considering the high reliability for SNV somatic mutations, we inferred the evolutionary relationships among GBC, LG-, and HG-BilIN based on their SNV mutations primarily. To ensure the accuracy of the evolutionary trees, we employed two independent methods, Treeomics^29^ and MEGAX^30^, and observed very consistent tumor sample phylogenetic topology for all 11 patients (Methods). The trees could be classified into two groups: (i) the BilIN-independent group (6 patients) in which GBC split before the common ancestor of LG- BilIN and HG-BilIN, and GBC evolved more independently from the two BilIN tumors (Fig. 3a); and (ii) the BilIN-dependent group (5 patients) in which GBC split after the common ancestor of LG-BilIN and HG-BilIN, and GBC was clustered more closely with HG-BilIN in two patients and with LG-BilIN in three patients (Fig. 3b). To assess the classification robustness, we performed the same analysis on the phylogenetic trees inferred from gene-level SCNA values or large-scale loss-of heterozygosity (LOH) status, respectively, and found that the mutation-tree group classification indeed represent the consensus assignment in all the cases (Supplementary Data 4). Interestingly, the two young patients (P03 and P11, diagnosed at 38 years) were classified into separate groups (P03: BilIN-dependent; P11: BilIN-independent). Specifically, GBC, LG- BilIN, and HG-BilIN from P03 only shared one potential driver mutation (*CTNNB1*.pT41I), while GBC in P11 had one *SMARCA4* mutation, and HG-BilIN in P11 harbored one *ARID2* mutation. These results suggest two coexisting evolutionary paths for GBC development: the BilIN-dependent model is more aligned with the traditional adenoma/dysplasia-carcinoma sequence model, wherein benign tumors, LG-/HG-BilIN, serve as the precursors of GBC; and the BilIN-independent model that suggests an earlier diverged origin of GBC. Interestingly, although both the number of mutations and the age at diagnosis were very similar in the two groups (Fig. 3c, d), the number of mutations acquired in the common ancestor of LG-BilIN/HG-BilIN/GBC was significantly higher in the BilIN-independent group than in the BilIN-dependent group (mean, 150 vs. 36, Wilcoxon rank-sum test, p <0.05). These results suggest that the mutation load accumulated in the early stage of tumor development, rather than the total mutation load in GBC, affects the evolutionary path of GBC.

**Fig. 4:**
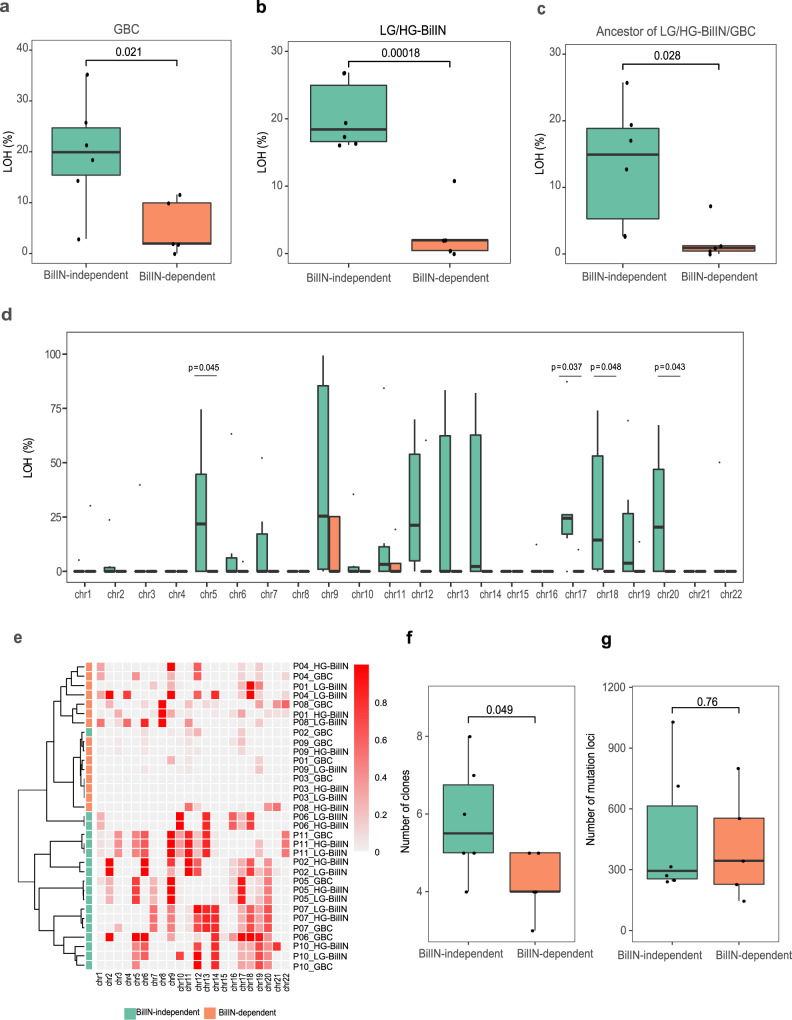
Comparison of the LOH and clonal evolution of tumors in the BilIN-independent and BilIN-dependent groups. **a** LOH percentage in GBC samples (BilIN-independent: *n* = 6; BilIN-dependent: *n* = 5). **b** LOH percentage in BilIN samples (BilIN-independent: *n* = 6; BilIN-dependent: *n* = 5). **c** LOH percentage shared by GBC, LG-BilIN, and HG-BilIN (BilIN-independent: *n* = 6; BilIN-dependent: *n* = 5). **d** Chromosome-level LOH shared by GBC, LG-BilIN, and HG-BilIN samples (BilIN-independent: *n* = 6; BilIN-dependent: *n* = 5). **e** A heatmap showing sample clustering patterns based on chromosome-wide LOH status. **f** The number of clones and **g** the number of mutation loci used in the clonal evolution analysis. The middle line in the box is the median, the bottom, and top of the box are the first and third quartiles, and the whiskers extend to 1.5× interquartile range of the lower and the upper quartiles, respectively. Raw *p*-values based on the two-tailed Student’s *t*-test are shown.

To identify other driving factors affecting the evolutionary paths of GBC, we compared genomic features of GBC samples between the two groups but did not detect any significant differences in specific mutated drivers and MSI. LOH is a common occurrence in cancer, and the accumulation of LOH can be associated with carcinogenesis of the gallbladder^31,32^. Interestingly, we observed that GBC samples in the BilIN-independent group showed a higher proportion of LOH than those in the BilIN-dependent group (mean, 20% vs. 5%, *t*-test, *p* = 0.021, Fig. 4a). This observation raised two possible evolutionary scenarios for the BilIN-independent group: (i) large-scale LOH events occurred in the common ancestor of LG-BilIN/HG-BilIN/GBC, which then led to an early split of GBC; or (ii) after the divergence from LG-BilIN /HG-BilIN, LOH events independently occurred in the GBC lineage. To identify the more likely scenario, we compared the LOH status of LG-BilIN /HG-BilIN and found that they showed the same pattern as GBC samples (mean, 20% vs. 3%, *t*-test, *p* = 1.8 × 10^−4^, Fig. 4b). We further compared the LOH status of the common ancestor of LG-BilIN/HG-BilIN/GBC (by considering LOH regions shared among the three tumors) and observed that LOH events were significantly more extensive in the BilIN-independent group than in the BilIN-dependent group (mean, 13% vs. 2%, *t*-test, *p* = 0.028, Fig. 4c). Finally, to rule out the possibility that LOH regions may affect the mutation-based tree construction, we rebuilt the phylogenetic trees based only on the mutations in non-LOH regions and observed the same patterns (Supplementary Fig. 5). When examining the LOH status of the common ancestor of LG-BilIN/HG-BilIN/GBC samples at the chromosome level, we found that chr5, chr17, chr18, and chr20 showed the most striking differences (*t*-test, *p* < 0.05, Fig. 4d). Indeed, the clustering pattern of gallbladder tumors based on their chromosome-level LOH status largely recaptured the tumor group classification based on mutation-based tree topologies (Fig. 4e). To gain more insights into the clonal evolution, we estimated the purity and ploidy of GBC/LG-BilIN/HG-BilIN samples using FACETS^33^ and then inferred the number of clones for tumor evolution in each patient using PyClone^34^. We found that tumors in the BilIN-independent group contained significantly more clones than those in the BilIN-dependent group (*t*-test, *p* = 0.049, Fig. 4f), although they did not contain more somatic mutations (*t*-test, *p* = 0.76, Fig. 4g). We also investigated the possible seeding patterns for GBC, based on inferred cancer cell fractions (CCF) of mutations^35^ and found that all 11 GBC cases were polyclonal seeding (Supplementary Data 5).

**Fig. 5:**
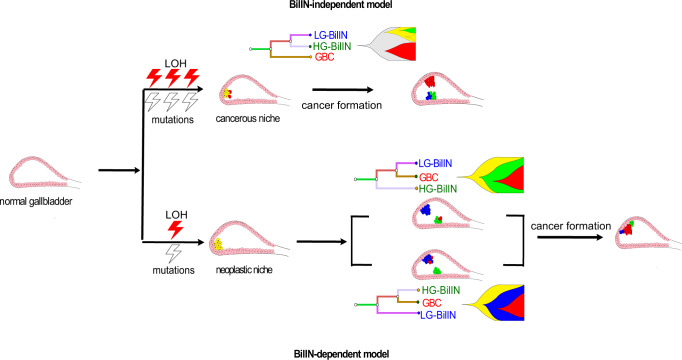
Schematic representation of a proposed evolutionary model of GBC with coexisting LG-BilIN and HG-BilIN. In the BilIN-independent group, GBC tends to split earlier and evolve more independently from LG-BilIN and HG-BilIN in the cancerous niche that harbors extensive LOH and mutation events. In the BilIN-dependent group, the neoplastic niche needs a step-wise progression to achieve the malignant state.

Based on these results, we proposed an evolutionary model of GBC development in which the extent of LOH events and somatic mutations accumulated at the initial stage of gallbladder tumor development is the key to determine the subsequent path of carcinoma evolution (Fig. 5). In the BilIN-independent group, extensive LOH and mutation events occur first, leading to a “cancerous” niche in the common ancestor of LG-BilIN/HG-BilIN/GBC. Given the acquired cancer potentiality of the cancerous niche, GBC then tends to split earlier and evolve more independently from LG-BilIN and HG-BilIN, and there are more clones in the microevolutionary process. In the BilIN-dependent group, fewer LOH events and somatic mutations occur initially, resulting in a “neoplastic” niche in the common ancestor. The neoplastic niche needs a step-wise progression to achieve the malignant form, and the sprouting of tumors is based on dysplastic or neoplastic cells. From this niche, GBC tends to split later and evolve through an intermediate stage with LG-BilIN or HG-BilIN, and there are fewer clones.

## Discussion

GBC is a malignancy with poor overall survival, and the molecular research on its pathogenesis and carcinogenesis is relatively rare. Like most epithelial cancer types, GBC is preceded by a series of precancerous histological and molecular changes that evolve possibly over a period of several decades^18^. Adenoma and hyperplastic changes progressing to dysplasia are often assumed to be the usual form of GBC^36^, offering the opportunity to elucidate cancer pathogenesis and natural history in this disease. Through a genome-wide characterization of somatic alterations in a series of coexisting LG-BilIN, HG-BilIN, and GBC, we have deciphered a detailed molecular portrait of how GBC evolves in the context of gallbladder polyps. The simultaneous analysis of different gallbladder tumor types (both benign and malignant) from the same patients allowed us to reconstruct the evolutionary history with high confidence, distinguishing our study from previous investigations on GBC^7,19,20^ that were based on comparisons between precancerous and carcinomatous lesions from separate patient populations.

Our mutational analysis highlights that all LG-BilIN, HG-BilIN, and GBC are an inevitable consequence of aging and mutation accumulation, characterized by an age-related mutational signature (S1) as a major component observed in all the tumors. Putting this finding in the context of the median age of onset of these pathomorphological changes is informative. Gallbladder polyps with symptomatic cholecystitis seldom appear before the age of 40, the median age for detection of adenoma (LG-BilIN) is 45 years, and that for HG-BilIN is 50 years, while the median age of GBC diagnosis is 71 years^11,13,37^. It can thus be speculated that the cancerous lesions start as small clusters of neoplastic epithelial cells at an early stage that then increases in size and number over the years. At the time of cancerous transition, mutation-driven uncontrollable proliferation usually selects the clones with oncogenic potential. We observed several recurrent driver mutations in GBC, including *TP53*, *KRAS*, *ARID2*, *SMAD4*, *PI3KCA*, and *ERBB3*, similar to a previous study^38^. Notably, our results suggest a critical role of *CTNNB1* mutations in the cellular transformation from normal cell growth into a state of uncontrollable proliferation. Previous studies reported that adenoma or LG-BilIN expresses significantly higher β-catenin than GBC, and 62.5% of LG-BilIN samples harbored mutations in *CTNNB1* exon 3, but this rate was only 4.8% in the GBC^39,40^. In our cohort, 5 of the 11 patients harbored *CTNNB1* mutations; in 4 of these 5 cases, GBC and its adjacent LG-BilIN shared the same mutations in *CTNNB1*, suggesting that *CTNNB1* mutations play a more active role in the formation of carcinoma in the context of adenoma/dysplasia than GBC alone.

Two major developmental paths have been suggested for GBC: (i) de novo development when only GBC is present; and (ii) the adenoma/dysplasia-carcinoma sequence model when GBC coexists with LG-BilIN or HG-BilIN^2^. However, it remains unclear how the GBC developmental paths are determined and whether LG-BilIN or HG-BilIN represents a major transition state in the latter model. Through a comprehensive, multilayer analysis of the cancer genome, our study suggests that the relationship between GBC and adjacent LG-BilIN/HG-BilIN can be divided into two evolutionary paths, the BilIN-independent path, and the BilIN-dependent path. The BilIN-independent model is more aligned with the de novo development path wherein the carcinoma initiates before the divergence of LG-BilIN and HG-BilIN, and evolves more independently. In contrast, the BilIN-dependent divergence model is similar to the adenoma/dysplasia-carcinoma sequence where carcinoma evolves from LG-BilIN or HG-BilIN through a step-wise progressive manner. The key determinant is the accumulation of LOH and somatic mutation events in the initial stage that is sufficient to form a cancerous niche for subsequent GBC development. Interestingly, besides somatic mutations, we uncovered contrasting patterns of LOH events between the two evolutionary paths, which highlights the importance of losing functional tumor suppressor genes in the niche-defining process. Among individual chromosomes enriched for LOH events that presumably promote the cancerous niche, LOH on chr.5q and chr.7p have been associated with early carcinogenic changes of the gallbladder^31^, and importantly, LOH events are rare in the normal gallbladder^32^. Clinically, our results suggest that proactive intervention needs to focus on identifying patients with LOH-rich gallbladder tissue through noninvasive detection methods, so that earlier or more aggressive cholecystectomy may be adopted as a primary prevention approach for GBC. Furthermore, our results confirm that both dysplasia-carcinoma sequence (HG-BilIN-GBC) and adenoma-carcinoma sequence (LG-BilIN-GBC) exist in the GBC development. We did not observe significant bias towards LG-BilIN or HG-BilIN, despite the small sample size of our BilIN-dependent group (HG-BilIN-GBC, *n* = 2; LG-BilIN-GBC, *n* = 3). It remains unclear whether residual dysplasia at the cystic duct margin after cholecystectomy is associated with an increased incidence of recurrent carcinoma^41–43^. Thus, timely detection of GBC with neoplastic niches is critical in deciding the need for a BilIN-free surgical margin.

One limitation of this study is the representativeness of our cases. Since the cases (carcinomas coexisting with both adenoma and BilIN) surveyed are rare among gallbladder cancer with a prevalence from 1.8% to 6.4%^36,44,45^, further efforts are required to examine the evolutionary paths of more common gallbladder cancer. Other limitations may be the relatively small cohort size and the selection of manual sampling locations. To mitigate these, we have made extensive efforts in searching for such cases from a large tumor tissue bank, and have performed a careful pathological examination of all the sections of the resected gallbladder. We also ensured that every normal control sample was tumor-, adenoma-, and dysplasia-free mucosa, thereby removing the potential bias in phylogenetic tree inference. The mutations in cancer driver genes identified in our cohort are similar to previous findings, supporting their representativeness^38^. Further efforts are needed to confirm our evolutionary model using independent cohorts, especially prospective studies with sequential samplings from the normal gallbladder, LG-BilIN, emerging HG-BilIN, and GBC formation, over the disease course. The mechanisms through which different LOH events and mutation loads (high vs. low) could drive normal gallbladder mucosa into distinct states of gallbladder cancerous/neoplastic “niche” remain unclear. Further multi-omics characterization of epigenetic alterations, aneuploidy, or transcriptomes will provide more insights into these oncogenic transformations.

## Methods

### Patient recruitment and sample cohort

All samples were collected with the approval of the Institutional Review Board (IRB) from Peking Union Medical College Hospital (PUMCH), Beijing, China. Informed and written consent was obtained from each patient. We enrolled 11 patients who underwent surgical resection of gallbladder polyps at PUMCH from April 2008 to December 2016 and had pathologically confirmed geographically coexisting GBC, LG-BilIN, and HG-BilIN lesions, according to the 2019 WHO classification of tumors of the digestive system^46^. For LG-BilIN, we only included gallbladder adenoma with low-grade dysplasia, referring to the International Classification of Diseases for Oncology (ICD-O) code as 8503/0. The HG-BilIN indicated the regions with high-grade dysplasia or carcinoma in situ (CIS), referring to ICD-0 code as 8503/2. All GBC lesions were T1 stage tumors, including tumors invaded lamina propria and tumors infiltrating muscle layer. The inclusion of samples required GBC, LG-BilIN, HG-BilIN, and normal gallbladder tissues for each patient. Independent pathologists evaluated the pathology of all the samples in this study and obtained consistent results. To minimize the cancerization effect on the normal samples, we only included normal gallbladder tissues in a paraffin block that contained no cancer. All clinical data were obtained from the hospital records.

### Whole-exome sequencing

Serial consecutive 5-μm-thick sections were cut from representative deparaffinized blocks and stained with hematoxylin. Multiple pathologists independently confirmed the diagnosis, different histological features, and adequate cellularity. Four components, GBC, LG-BilIN, HG-BilIN, and normal tissue, were separately laser-microdissected using a Leica CTR 6000 Microsystem (Wetzlar, Germany) from the slides of each patient. Genomic DNA (gDNA) was extracted using the QIAamp DNA FFPE Tissue Kit (Qiagen, Germany) according to the manufacturer’s protocol. Then, 0.1 µg DNA was sheared into 200–300 bp fragments using a Covaris sonicator (Covaris, MA, USA). The resulting DNA fragments were repaired and 3’ A-tailed. Adaptors were ligated to both ends of the fragments, followed by size selection. Size-selected fragments were amplified via polymerase chain reaction (PCR). Exome capture was performed using SureSelect Human All Exon V6 (Agilent) according to the manufacturer’s protocol, followed by PCR amplification. Libraries were sequenced on Illumina NovaSeq.

### Analysis of somatic mutations

Whole-exome sequencing read pairs were trimmed, and only read pairs with <3% N bases and >50% high-quality bases were kept for subsequent analyses. The resulting high-quality reads were aligned to the human reference genome (Homo_sapiens_assembly19) using Burrows-Wheeler Aligner (0.7.17)^47^. To improve the alignment accuracy, we used Genome Analysis Toolkit (GATK, version 3.8.1)^48^ to process BAM files, including marking duplicates, base quality recalibration, and local realignment around high-confidence insertions and deletions. Based on ~7000 high-frequency SNP sites, the identical call rate among GBC, LG-BilIN, HG-BilIN, and normal tissues was >95% using BAM-matcher^49^, confirming that these lesions were indeed from the same patients. We used the variant calling pipeline developed by TCGA MC3 project^22^. Briefly, this pipeline employed five callers to call SNV mutations, and three callers to identify small indels, with detailed annotation. We only focused on SNV mutations and indels meeting the following criteria: (1) site depth ≥10× in both normal and tumor samples; (2) supported by at least two callers; (3) located in regions targeted by WES probes. Since all the GBC patients were diagnosed at T1, to make a fair comparison with other cancer types, we employed a mutation dataset of ~1200 stage-I samples from 16 TCGA cancer types that were generated with the same bioinformatics pipeline. To calculate the TMB values, we only used missense/nonsense mutations or ORF-shift mutations in the overlapped targeted regions in this study with those defined in the TCGA MC3 project. For the GBC/LG-BilIN/HG-BilIN analysis, we further reconciled their mutation calls from the three samples of the same patient to increase the sensitivity of mutation detection. Specifically, for a specific mutation identified in only one or two samples in a patient, we rescued it in the remaining sample(s) of the same patient even if only one caller supported the mutation. We used MANTIS^24^ to call the MSI status for each sample based on 2539 loci from the mSINGS package^50^. For mutational signatures, we employed deconstructSigs^51^ to compute the relative contributions of the 30 known mutational signatures defined by the COSMIC database (version 2)^23^. We also repeated the mutational signature analysis based on COSMIC mutational signature version 3. To identify potential driver mutations, we examined the mutation status of driver genes identified through TCGA PanCanAtlas across 33 cancer types^26^, including missense mutations, nonsense mutations, splice sites, and nonstop mutations.

### Experimental validation of somatic mutations

Using the same genomic DNA subjected to WES, we performed Sanger sequencing on 44 SNV mutations with VAF >10% from four patients. We designed universally tagged primers (Sangon Biotech Co., Ltd., Shanghai) using Primer3Plus^52^ and compared them with those from public databases to avoid SNPs and nonspecific amplification. Supplementary Data 3 lists the primer sequences.

### Analysis of somatic copy-number alteration and loss of heterozygosity

Based on paired tumor-normal WES data, we first identified SCNAs using CNVkit (v0.9.3)^53^ with default parameters. Then we generated the pooled segmentation from all 33 samples of 11 patients using GISTIC2^54^, which applied both low-level (cutoff, +/–1) and high-level (cutoff, +/–2) thresholds to define the gene-level SCNAs. To identify potential SCNA drivers, we focused on a well-defined set of genes comprising frequently amplified oncogenes or deleted tumor suppressor genes^55^. We performed the LOH analysis using the SNP-pipeline followed by FACETS^33^ based on WES data. The germline information was generated by HaplotypeCaller from GATK3.8^48^.

### Phylogenetic tree construction

To infer the evolutionary relationships between GBC, LG-BilIN, and HG-BilIN samples from the same patients, we employed Treeomics^29^ and the maximum parsimony method from MEGAX^30^. Based on the status of SNV mutations, we obtained consistent phylogenetic topologies in 10 of the 11 patients. For the remaining patient, we obtained a consistent phylogenetic tree when including mutations detected outside the exome-targeted regions. We also performed the phylogenetic tree reconstruction based on gene-level SCNA values and large-scale LOH status as described above, respectively (Supplementary Data 4). According to the phylogenetic trees, we classified the 11 patients into two groups based on whether GBC split before or after the common ancestor of LG-BilIN and HG-BilIN: one group was labeled as BilIN-independent divergence (six patients), and the other was labeled as BilIN-dependent divergence (five patients). To remove the potential bias of LOH regions on mutation calling, we repeated the tree constructions using only the mutations in the non-LOH regions.

### Analysis of clonal evolution

We employed PyClone^34^, a statistical model based on a Bayesian clustering method, to infer the cancer cell fractions (CCF) for each SNV mutation with a Beta Binomial emission. The purity and ploidy for most samples were successfully estimated by FACETS based on segmented genome^33^ except for those from P03 and P07, whose genome showed few alterations. The samples from these two patients were further estimated by Control-FREEC^56^ with a ploidy set to 2. For each SNV mutation, we extracted its major and minor copy numbers generated by FACETS. Based on PyClone estimation, we only counted those clones (mutation clusters) with ≥10 mutations. To infer the tumor seeding pattern for GBC patients, we compared the CCFs between sample pairs and calculated the Jaccard similarity index (JSI) for each pair as previously described^35^ and the JSI cutoff of 0.3 to distinguish monoclonal versus polyclonal seeding.

### Reporting summary

Further information on research design is available in the Nature Research Reporting Summary linked to this article.

## Supplementary information

Supplementary Information

Description of Additional Supplementary Files

Supplementary Data 1-5

Reporting Summary

## Data Availability

The raw WES data used in this study are available in the Genome Sequence Archive (GSA) database under accession code HRA00029 and the European Genome-Phenome Archive (EGA) database under the accession code EGAS00001005402. The person who wants to access the data can contact The Data Access Committee (DAC) listed at the websites. The access applications should be sent by email with a detailed introduction on the research intended to carry out based on the data. Somatic mutation data are also available in the European Variation Archive (EVA) database under the accession code PRJEB44269. Source data are available as a Source Data file. The remaining data are available within the Article and Supplementary Information. Source data are provided with this paper.

## Source data

Source Data

## References

[1] Varshney S, Butturini G, Gupta R (2002). Incidental carcinoma of the gallbladder. Eur. J. Surg. Oncol..

[2] Wistuba II, Gazdar AF (2004). Gallbladder cancer: lessons from a rare tumour. Nat. Rev. Cancer.

[3] Hundal R, Shaffer EA (2014). Gallbladder cancer: epidemiology and outcome. Clin. Epidemiol..

[4] Goetze TO, Paolucci V (2010). Adequate extent in radical re-resection of incidental gallbladder carcinoma: analysis of the German Registry. Surg. Endosc..

[5] Heitz L, Kratzer W, Grater T, Schmidberger J, group ES (2019). Gallbladder polyps - a follow-up study after 11 years. BMC Gastroenterol..

[6] Inui K, Yoshino J, Miyoshi H (2011). Diagnosis of gallbladder tumors. Intern. Med..

[7] Meirelles-Costa AL (2010). Are histological alterations observed in the gallbladder precancerous lesions?. Clin. (Sao Paulo).

[8] Kim YT (2001). Genetic alterations in gallbladder adenoma, dysplasia and carcinoma. Cancer Lett..

[9] Feng Z (2011). The risk factor of gallbladder cancer: hyperplasia of mucous epithelium caused by gallstones associates with p16/CyclinD1/CDK4 pathway. Exp. Mol. Pathol..

[10] Kozuka S, Tsubone N, Yasui A, Hachisuka K (1982). Relation of adenoma to carcinoma in the gallbladder. Cancer.

[11] Sun Y, Yang Z, Lan X, Tan H (2019). Neoplastic polyps in gallbladder: a retrospective study to determine risk factors and treatment strategy for gallbladder polyps. Hepatobiliary Surg. Nutr..

[12] Fearon ER, Vogelstein B (1990). A genetic model for colorectal tumorigenesis. Cell.

[13] Lee SR, Kim HO, Shin JH (2019). Reasonable cholecystectomy of gallbladder polyp - 10 years of experience. Asian J. Surg..

[14] Katabi N (2010). Neoplasia of gallbladder and biliary epithelium. Arch. Pathol. Lab. Med..

[15] Vicent S (2019). Experimental models to unravel the molecular pathogenesis, cell of origin and stem cell properties of cholangiocarcinoma. Liver Int..

[16] Pai RK, Mojtahed K, Pai RK (2011). Mutations in the RAS/RAF/MAP kinase pathway commonly occur in gallbladder adenomas but are uncommon in gallbladder adenocarcinomas. Appl. Immunohistochem. Mol. Morphol..

[17] Itoi T (1996). APC, K-ras codon 12 mutations and p53 gene expression in carcinoma and adenoma of the gall-bladder suggest two genetic pathways in gall-bladder carcinogenesis. Pathol. Int..

[18] Barreto SG, Dutt A, Chaudhary A (2014). A genetic model for gallbladder carcinogenesis and its dissemination. Ann. Oncol..

[19] Roa I (1996). Preneoplastic lesions and gallbladder cancer: an estimate of the period required for progression. Gastroenterology.

[20] Akita M (2019). Intracholecystic papillary neoplasms are distinct from papillary gallbladder cancers: a clinicopathologic and exome-sequencing study. Am. J. Surgical Pathol..

[21] Lazcano-Ponce EC (2001). Epidemiology and molecular pathology of gallbladder cancer. CA Cancer J. Clin..

[22] Ellrott K (2018). Scalable open science approach for mutation calling of tumor exomes using multiple genomic pipelines. Cell Syst..

[23] Alexandrov LB (2015). Clock-like mutational processes in human somatic cells. Nat. Genet.

[24] Kautto EA (2017). Performance evaluation for rapid detection of pan-cancer microsatellite instability with MANTIS. Oncotarget.

[25] Adnan Awad S (2020). Mutation accumulation in cancer genes relates to nonoptimal outcome in chronic myeloid leukemia. Blood Adv..

[26] Bailey MH (2018). Comprehensive characterization of cancer driver genes and mutations. Cell.

[27] Adzhubei, I., Jordan, D. M. & Sunyaev, S. R. Predicting functional effect of human missense mutations using PolyPhen-2. *Curr. Protoc. Hum. Genet.* **Chapter 7**, Unit7.20 (2013). 10.1002/0471142905.hg0720s76 PMC4480630 23315928

[28] Rebouissou S (2016). Genotype-phenotype correlation of CTNNB1 mutations reveals different ss-catenin activity associated with liver tumor progression. Hepatology.

[29] Reiter JG (2017). Reconstructing metastatic seeding patterns of human cancers. Nat. Commun..

[30] Kumar S, Stecher G, Li M, Knyaz C, Tamura K (2018). MEGA X: molecular evolutionary genetics analysis across computing platforms. Mol. Biol. Evol..

[31] Chang HJ, Kim SW, Kim YT, Kim WH (1999). Loss of heterozygosity in dysplasia and carcinoma of the gallbladder. Mod. Pathol.: Off. J. U. S. Can. Acad. Pathol., Inc..

[32] Jain K (2014). Sequential occurrence of preneoplastic lesions and accumulation of loss of heterozygosity in patients with gallbladder stones suggest causal association with gallbladder cancer. Ann. Surg..

[33] Shen R, Seshan VE (2016). FACETS: allele-specific copy number and clonal heterogeneity analysis tool for high-throughput DNA sequencing. Nucleic Acids Res..

[34] Roth A (2014). PyClone: statistical inference of clonal population structure in cancer. Nat. Methods.

[35] Hu Z, Li Z, Ma Z, Curtis C (2020). Multi-cancer analysis of clonality and the timing of systemic spread in paired primary tumors and metastases. Nat. Genet..

[36] Roa I, de Aretxabala X, Araya JC, Roa J (2006). Preneoplastic lesions in gallbladder cancer. J. Surgical Oncol..

[37] Szpakowski J-L, Tucker L-Y (2020). Outcomes of gallbladder polyps and their association with gallbladder cancer in a 20-year cohort. JAMA Netw. Open.

[38] Li M (2014). Whole-exome and targeted gene sequencing of gallbladder carcinoma identifies recurrent mutations in the ErbB pathway. Nat. Genet..

[39] Chang HJ, Jee CD, Kim WH (2002). Mutation and altered expression of beta-catenin during gallbladder carcinogenesis. Am. J. Surgical Pathol..

[40] Yanagisawa N, Mikami T, Saegusa M, Okayasu I (2001). More frequent beta-catenin exon 3 mutations in gallbladder adenomas than in carcinomas indicate different lineages. Cancer Res..

[41] Akki AS (2019). Systematic selective sampling of cholecystectomy specimens is adequate to detect incidental gallbladder adenocarcinoma. Am. J. Surgical Pathol..

[42] Bickenbach KA (2011). High-grade dysplasia of the cystic duct margin in the absence of malignancy after cholecystectomy. HPB: Off. J. Int. Hepato Pancreato Biliary Assoc..

[43] Moslim, M. A., Tang, A. & Morris-Stiff, G. Management of high-grade dysplasia of the cystic duct after cholecystectomy. *BMJ Case Rep.* **2017**, bcr2016218994 (2017). 10.1136/bcr-2016-218994 PMC5534645 28645922

[44] Adsay V (2012). Intracholecystic papillary-tubular neoplasms (ICPN) of the gallbladder (neoplastic polyps, adenomas, and papillary neoplasms that are >/=1.0 cm): clinicopathologic and immunohistochemical analysis of 123 cases. Am. J. Surg. Pathol..

[45] Lee SH (2010). [Histopathologic analysis of adenoma and adenoma-related lesions of the gallbladder]. Korean J. Gastroenterol..

[46] Nagtegaal ID (2020). The 2019 WHO classification of tumours of the digestive system. Histopathology.

[47] Li H, Durbin R (2009). Fast and accurate short read alignment with Burrows-Wheeler transform. Bioinformatics.

[48] DePristo MA (2011). A framework for variation discovery and genotyping using next-generation DNA sequencing data. Nat. Genet..

[49] Wang PP, Parker WT, Branford S, Schreiber AW (2016). BAM-matcher: a tool for rapid NGS sample matching. Bioinformatics.

[50] Salipante SJ, Scroggins SM, Hampel HL, Turner EH, Pritchard CC (2014). Microsatellite instability detection by next generation sequencing. Clin. Chem..

[51] Rosenthal R, McGranahan N, Herrero J, Taylor BS, Swanton C (2016). DeconstructSigs: delineating mutational processes in single tumors distinguishes DNA repair deficiencies and patterns of carcinoma evolution. Genome Biol..

[52] Untergasser A (2012). Primer3–new capabilities and interfaces. Nucleic Acids Res..

[53] Talevich E, Shain AH, Botton T, Bastian BC (2016). CNVkit: genome-wide copy number detection and visualization from targeted DNA sequencing. PLoS Comput. Biol..

[54] Mermel CH (2011). GISTIC2.0 facilitates sensitive and confident localization of the targets of focal somatic copy-number alteration in human cancers. Genome Biol..

[55] Zack TI (2013). Pan-cancer patterns of somatic copy number alteration. Nat. Genet.

[56] Boeva V (2012). Control-FREEC: a tool for assessing copy number and allelic content using next-generation sequencing data. Bioinformatics.

