# Supplementary material for: Genomic characterization of co-existing neoplasia and carcinoma lesions reveals distinct evolutionary paths of gallbladder cancer
Source: Nat Commun. 2021 Aug 6;12:4753. doi: 10.1038/s41467-021-25012-9 (PMC8346570; doi:10.1038/s41467-021-25012-9)
Supplement: Supplementary file 1 — Supplementary Information [file 41467_2021_25012_MOESM1_ESM.pdf]

Supplementary Information

Genomic characterization of co-existing neoplasia and carcinoma lesions reveals distinct evolutionary paths of gallbladder cancer

Lin et al.

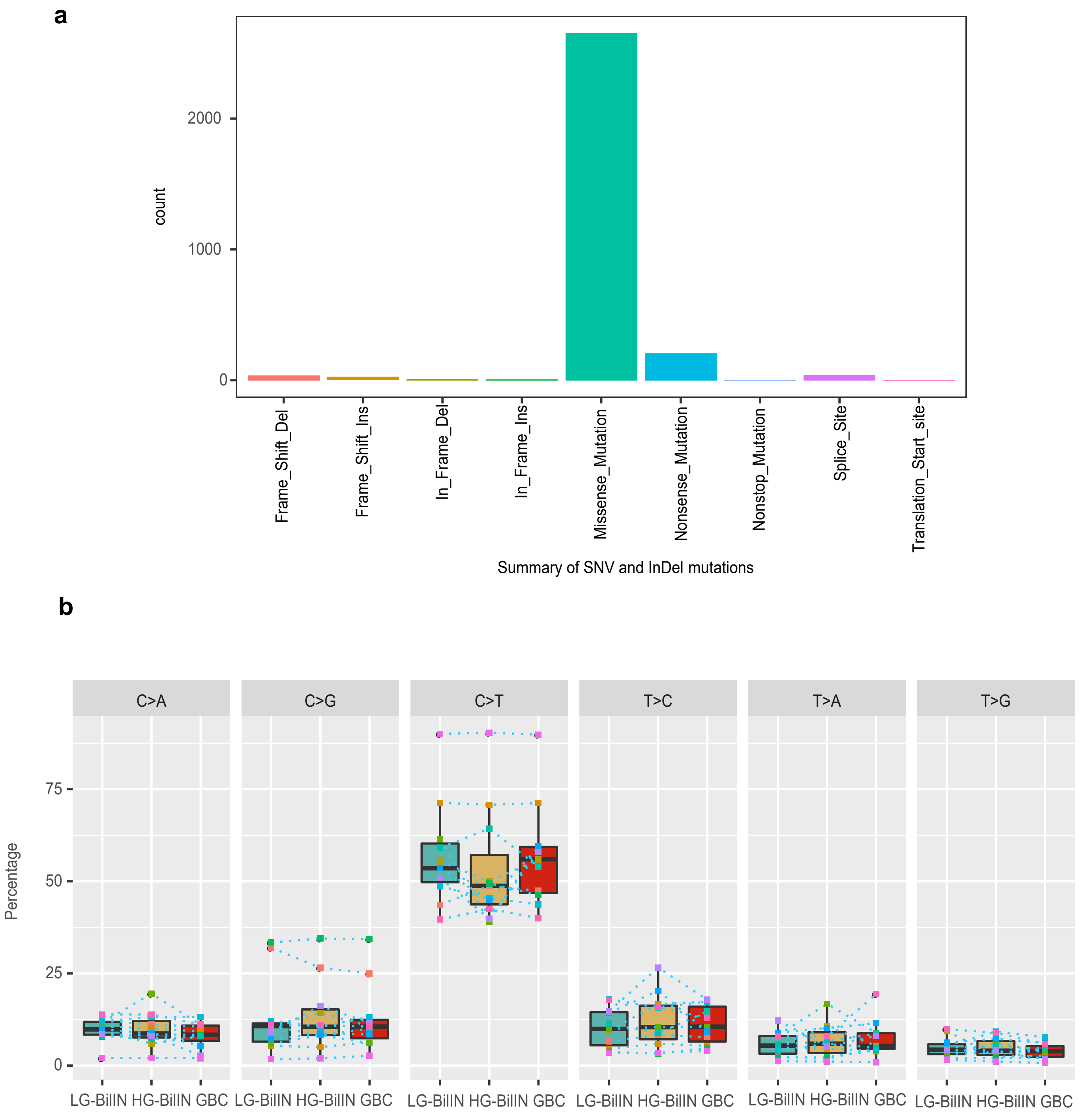

Supplementary Figure 1 | Summary of somatic mutations in gallbladder tumors
(a) Distribution of different types of somatic mutations. (b) Comparison of different types of substitutions in GBA, LG-BilIN and HG-BilIN (n = 11). The middle line in the box is the median, the bottom, and top of the box are the first and third quartiles, and the whiskers extend to 1.5× interquartile range of the lower and the upper quartiles, respectively.

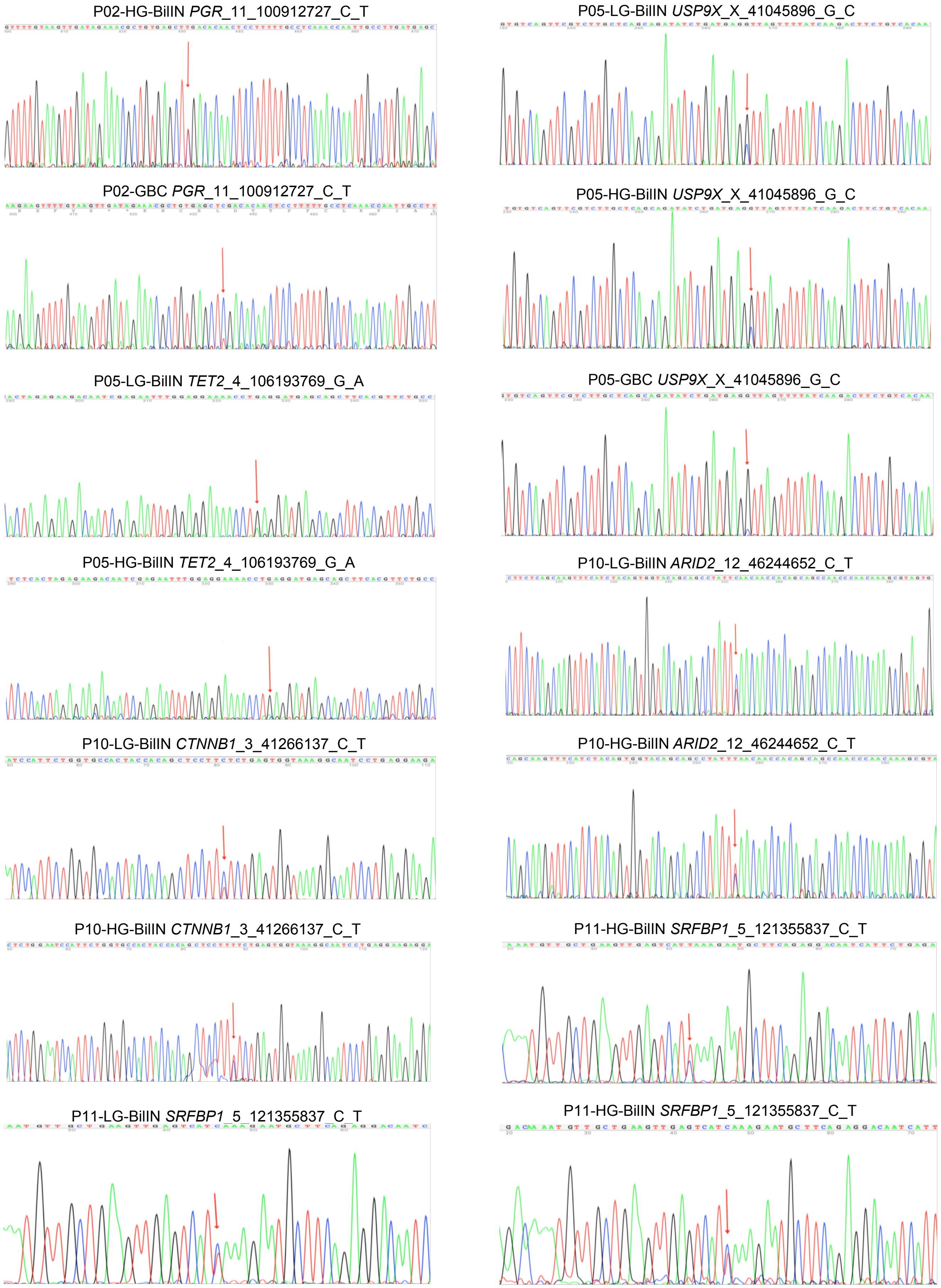

Supplementary Figure 2 | Representative mutational signals in Sanger-sequencing-based experimental validation

In each panel, the somatic mutation of interest is indicated by a red arrow.

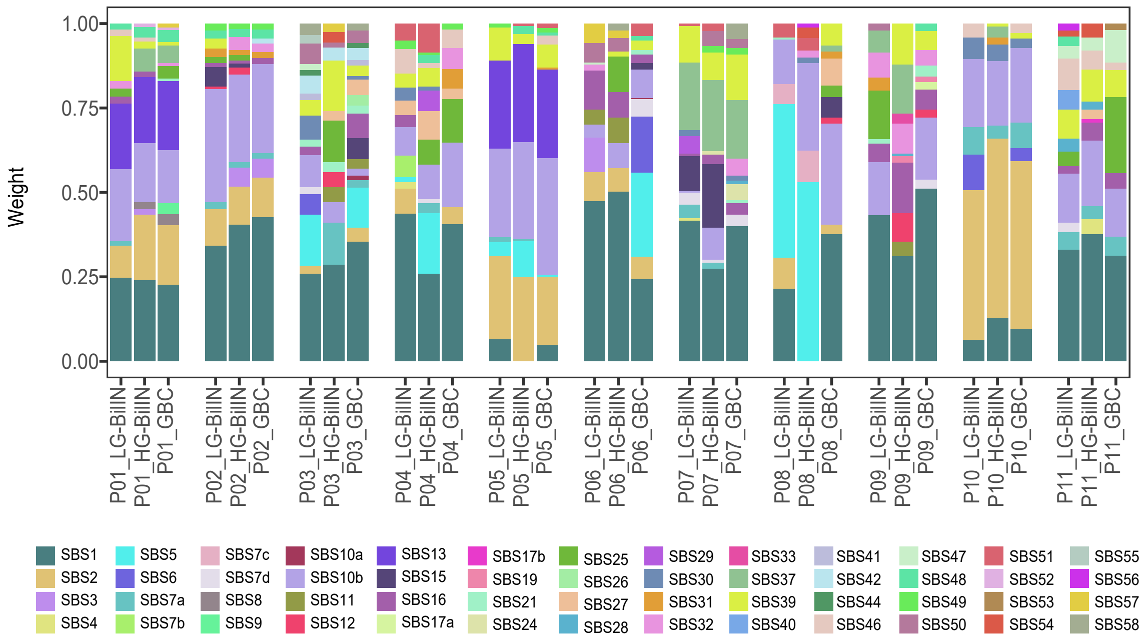

Supplementary Figure 3 | Signature deconvolution analysis based on COSMIC mutational signature v3

Relative contributions of different mutational signatures in tumor samples based on COSMIC mutational signature v3.

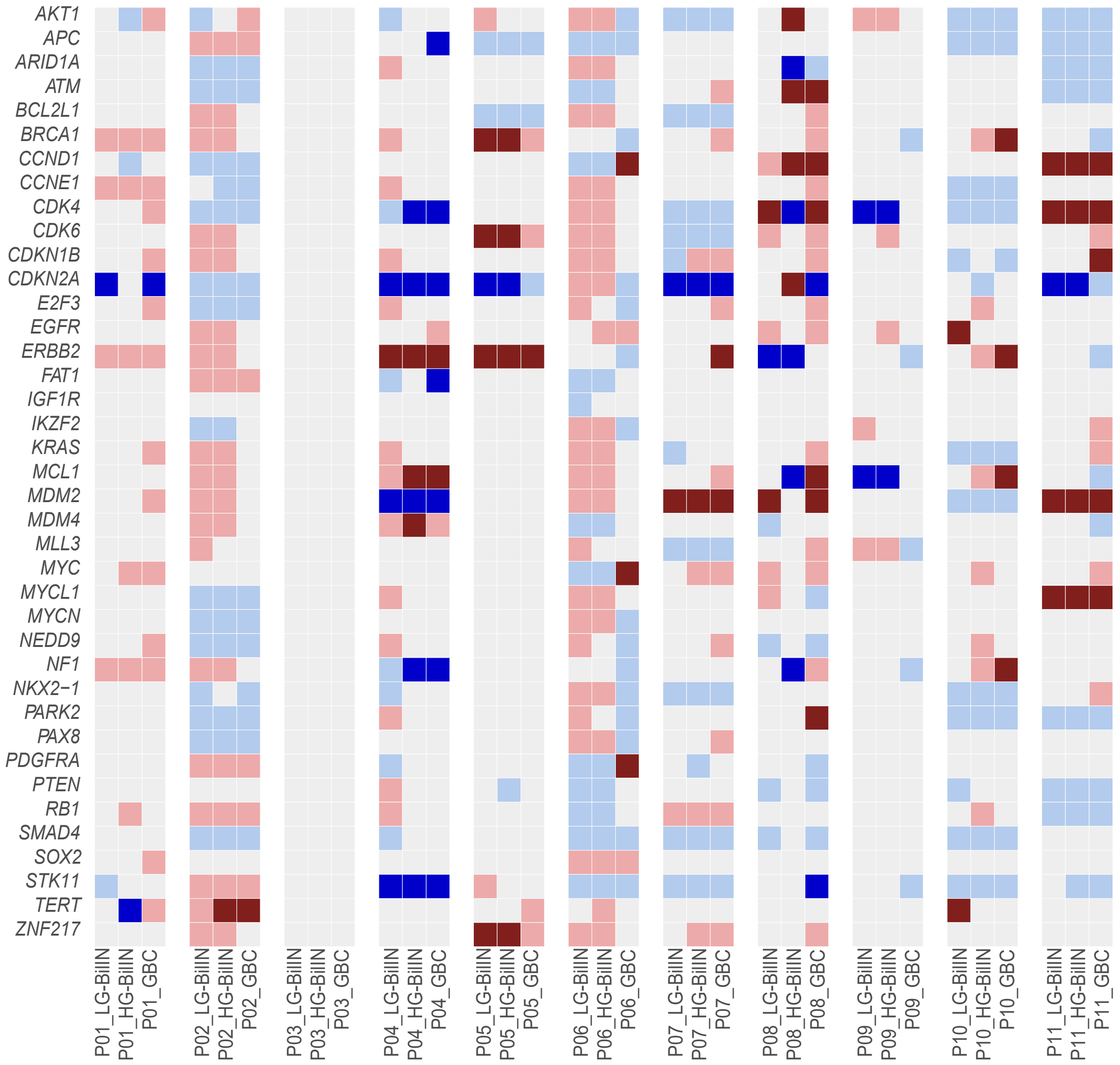

Supplementary Figure 4 | The landscape of SCNA driver candidates in BilIN-related gallbladder tumors
SCNA types are shown in different colors: dark red, high amplification; light red, low amplification; dark blue, deep deletion; light blue, shallow deletion.

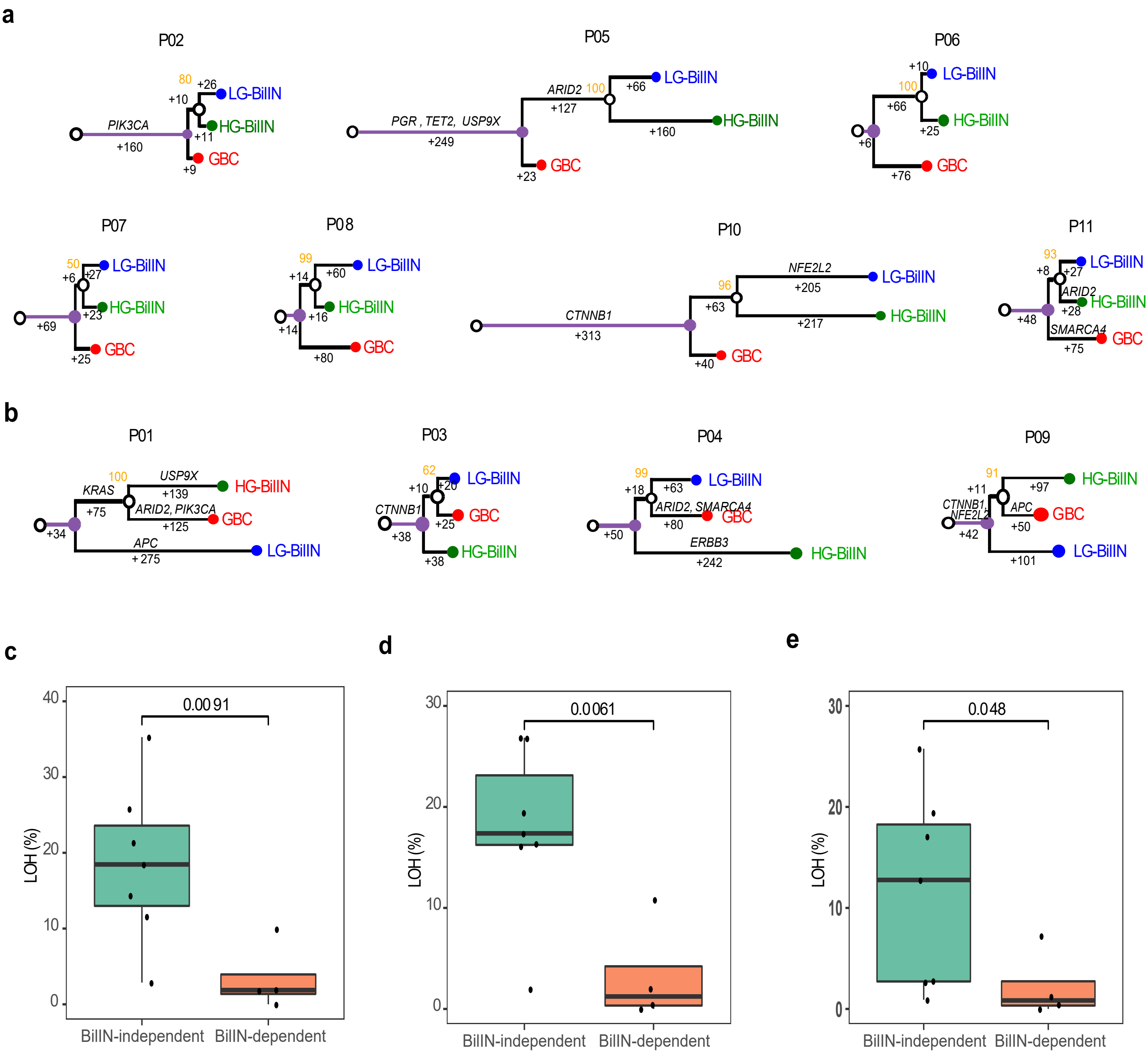

Supplementary Figure 5 | Comparison of the LOH status of tumor samples between the BilIN-independent and BilIN-dependent groups based on the new tree classification

(a, b) The phylogenetic trees inferred from mutations in non-LOH regions (a) BilIN-independent group (n = 7), and (b) BilIN-dependent group (n = 4). The branch lengths are proportional to the number of supporting mutations, which were labeled. Bootstrap values are shown in orange. Based on these trees, 10 of the 11 patients remained the same group classification, and only one patient (P08) switched groups. (c) LOH percentage in GBC samples (BilIN-independent group: 7; BilIN-dependent group: 4). (d) LOH percentage in BilIN samples (BilIN-independent group: 7; BilIN-dependent group: 4). (e) LOH percentage shared by GBC, LG-BilIN, and HG-BilIN (BilIN-independent group: 7; BilIN-dependent group: 4). The middle line in the box is the median, the bottom, and top of the box are the first and third quartiles, and the whiskers extend to 1.5 × interquartile range of the lower and the upper quartiles, respectively. Raw p values based on two-tailed Student's t-test are shown.
