# Supplementary material for: Genomic characterization of co-existing neoplasia and carcinoma lesions reveals distinct evolutionary paths of gallbladder cancer
Source: Nat Commun. 2021 Aug 6;12:4753. doi: 10.1038/s41467-021-25012-9 (PMC8346570; doi:10.1038/s41467-021-25012-9)
Supplement: Supplementary file 2 — Description of Additional Supplementary Files [file 41467_2021_25012_MOESM2_ESM.pdf]

Description of Additional Supplementary Files

File name: Supplementary Data 1

Description: The clinical and sample information of the gallbladder cancer patients included in this study.

File name: Supplementary Data 2

Description: Somatic mutations identified in this study.

File name: Supplementary Data 3

Description: Information about the mutations selected for experimental validation.

File name: Supplementary Data 4

Description: GBC evolutionary group assignment based on mutations, SCNAs or LOH.

File name: Supplementary Data 5

Description: Jaccard similarity scores for tumor samples in this study.
