# Supplementary material for: Genomic characterization of co-existing neoplasia and carcinoma lesions reveals distinct evolutionary paths of gallbladder cancer
Source: Nat Commun. 2021 Aug 6;12:4753. doi: 10.1038/s41467-021-25012-9 (PMC8346570; doi:10.1038/s41467-021-25012-9)
Supplement: Supplementary file 4 — Reporting Summary [file 41467_2021_25012_MOESM4_ESM.pdf]

nature research

Corresponding author(s): Han Liang

Last updated by author(s): 2021-5-2

Reporting Summary

Nature Research wishes to improve the reproducibility of the work that we publish. This form provides structure for consistency and transparency in reporting. For further information on Nature Research policies, see our Editorial Policies and the Editorial Policy Checklist.

Statistics

For all statistical analyses, confirm that the following items are present in the figure legend, table legend, main text, or Methods section.

n/a	Confirmed	
☐	☒	The exact sample size (*n*) for each experimental group/condition, given as a discrete number and unit of measurement
☐	☒	A statement on whether measurements were taken from distinct samples or whether the same sample was measured repeatedly
☐	☒	The statistical test(s) used AND whether they are one- or two-sided<br>*Only common tests should be described solely by name; describe more complex techniques in the Methods section.*
☐	☒	A description of all covariates tested
☐	☒	A description of any assumptions or corrections, such as tests of normality and adjustment for multiple comparisons
☐	☒	A full description of the statistical parameters including central tendency (e.g. means) or other basic estimates (e.g. regression coefficient) AND variation (e.g. standard deviation) or associated estimates of uncertainty (e.g. confidence intervals)
☐	☒	For null hypothesis testing, the test statistic (e.g. *F*, *t*, *r*) with confidence intervals, effect sizes, degrees of freedom and *P* value noted<br>*Give P values as exact values whenever suitable.*
☒	☐	For Bayesian analysis, information on the choice of priors and Markov chain Monte Carlo settings
☒	☐	For hierarchical and complex designs, identification of the appropriate level for tests and full reporting of outcomes
☐	☒	Estimates of effect sizes (e.g. Cohen's *d*, Pearson's *r*), indicating how they were calculated

Our web collection on statistics for biologists contains articles on many of the points above.

Software and code

Policy information about availability of computer code

Data collection	No software was used.
Data analysis	Burrows-Wheeler Aligner (0.7.17); Genome Analysis Toolkit (GATK, version 3.8.1); Treeomics and MEGA (version X); BAM-matcher; SignatureAnalyzer; MANTIS v1.0.3; MuSic v0.2; CNVkit v0.9.3; mSINGs(2014); GISTIC(v2.0). In-house R scripts were written in R v3.6.2.

For manuscripts utilizing custom algorithms or software that are central to the research but not yet described in published literature, software must be made available to editors and reviewers. We strongly encourage code deposition in a community repository (e.g. GitHub). See the Nature Research guidelines for submitting code & software for further information.

Data

Policy information about availability of data

All manuscripts must include a data availability statement. This statement should provide the following information, where applicable:

- Accession codes, unique identifiers, or web links for publicly available datasets
- A list of figures that have associated raw data
- A description of any restrictions on data availability

The sequencing data reported in this study have been deposited in the Genome Sequence Archive under the accession number HRA000229 in BIG Data Center, Beijing Institute of Genomics (BIG), Chinese Academy of Sciences, and are publicly accessible at http://bigd.big.ac.cn/gsa; and the European Variation Archive (EVA) database under the accession code PRJEB44269 (https://www.ebi.ac.uk/eva/).

Field-specific reporting

Please select the one below that is the best fit for your research. If you are not sure, read the appropriate sections before making your selection.

☒ Life sciences ☐ Behavioural & social sciences ☐ Ecological, evolutionary & environmental sciences

For a reference copy of the document with all sections, see nature.com/documents/nr-reporting-summary-flat.pdf

Life sciences study design

All studies must disclose on these points even when the disclosure is negative.

Sample size	n = 33. This is the largest size for such a comparative analysis. The power of detecting a feature with a fold change > 2 is 0.9 at FDR = 0.05
Data exclusions	No data exclusion
Replication	This is a patient-cohort based analysis, and each tumor sample is independent. So there is no replication.
Randomization	No. This is a retrospective patient-cohort based analysis. No clinical response or survival data were used in the analysis.
Blinding	No. This is a retrospective patient-cohort based analysis. No clinical response or survival data were used in the analysis.

Reporting for specific materials, systems and methods

We require information from authors about some types of materials, experimental systems and methods used in many studies. Here, indicate whether each material, system or method listed is relevant to your study. If you are not sure if a list item applies to your research, read the appropriate section before selecting a response.

Materials & experimental systems

n/a	Involved in the study	
☒	☐	Antibodies
☒	☐	Eukaryotic cell lines
☒	☐	Palaeontology and archaeology
☒	☐	Animals and other organisms
☐	☒	Human research participants
☒	☐	Clinical data
☒	☐	Dual use research of concern

Methods

n/a	Involved in the study	
☒	☐	ChIP-seq
☒	☐	Flow cytometry
☒	☐	MRI-based neuroimaging

Human research participants

Policy information about studies involving human research participants

Population characteristics	The patients underwent surgical resection of gallbladder polyps at PUMCH from April 2008 to December 2016 and had pathologically confirmed geographically co-existing GBC, LG-BilIN, and HG-BilIN lesions, according to the 2019 WHO classification of tumours of the digestive system.
Recruitment	The patients were recruited at Peking Union Medical College Hospital, China.
Ethics oversight	All samples were collected with the approval of the Institutional Review Board (IRB) from Peking Union Medical College Hospital (PUMCH), Beijing, China. Informed and written consent was obtained from each patient.

Note that full information on the approval of the study protocol must also be provided in the manuscript.
